# Monolith immuno-affinity enrichment liquid chromatography tandem mass spectrometry for quantitative protein analysis of recombinant bovine somatotropin in serum

**DOI:** 10.1007/s00216-015-8775-2

**Published:** 2015-06-16

**Authors:** Nathalie G. E. Smits, Marco H. Blokland, Klaas L. Wubs, Merel A. Nessen, Leen A. van Ginkel, Michel W. F. Nielen

**Affiliations:** RIKILT Wageningen UR, P.O. Box 230, 6700 AE Wageningen, The Netherlands; Laboratory of Organic Chemistry, Wageningen University, Dreijenplein 8, 6703 HB Wageningen, The Netherlands

**Keywords:** Liquid chromatography, Mass spectrometry, Immuno-affinity enrichment, Monolith micro-column, Recombinant bovine somatotropin, bST

## Abstract

The use of recombinant bovine somatotropin (rbST) to enhance milk production is approved in several countries, but it is prohibited in the European Union. According to EU legislation, it is necessary to confirm positive screening results prior to enforcement. Although adequate screening assays are available nowadays, development of liquid chromatography tandem mass spectrometry (LC-MS/MS) confirmatory methods to detect low levels of rbST is still a challenge. Here, we present a novel approach using immuno-affinity enrichment on monolithic micro-columns in combination with state-of-the-art ultra-high pressure LC-MS/MS (UHPLC-MS/MS) detection. The developed approach enables detection and confirmation of rbST in serum at a decision limit (CCα) concentration of 0.8 ng mL^−1^. Furthermore, the method is easy to handle, robust and reproducible. We successfully applied the confirmatory method to serum samples from rbST treated cows that were found suspect after immunoassay-based screening. The use of rbST could be confirmed over 1 week after treatment, and the developed method demonstrated the sensitivity needed for effective control.

**Graphical Abstract:**
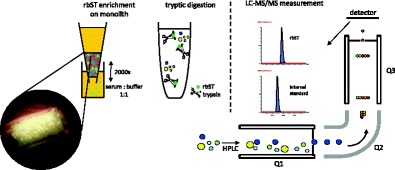
Graphical summary of the workflow, for serum preparation, enrichment with monolith microcolumns and LC-MS/MS measurement of rbST

Graphical summary of the workflow, for serum preparation, enrichment with monolith microcolumns and LC-MS/MS measurement of rbST

## Introduction

**Fig. 1:**
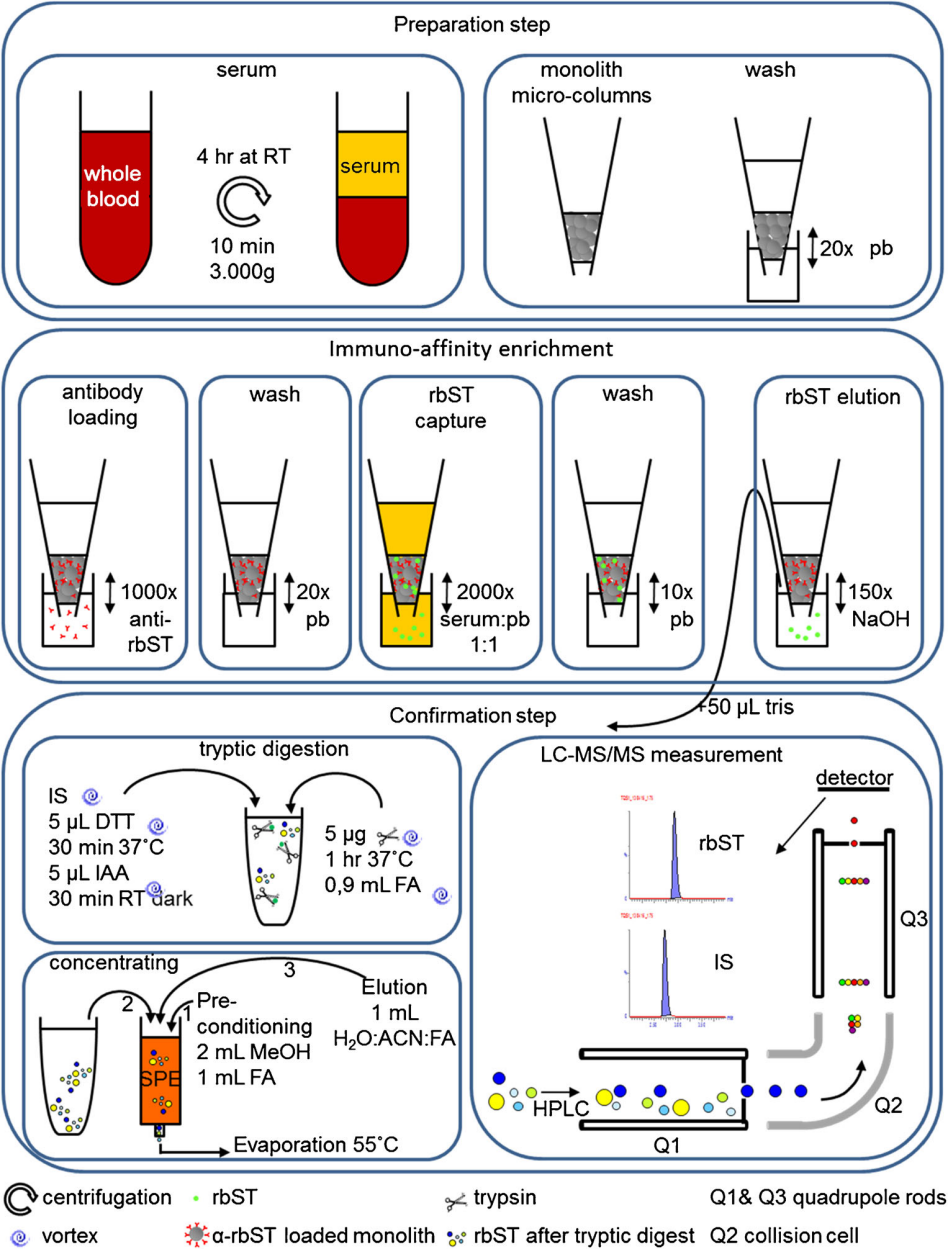
Workflow for serum preparation, enrichment with monolith micro-columns and LC-MS/MS measurement of rbST. *SPE* solid phase extraction, *pb* phosphate buffer, *RT* room temperature, *FA* formic acid, *ACN* acetonitrile

Already in the 1930s, extracts of pituitary glands containing endogenous bST were used to increase milk yield [1, 2]. Widespread application, however, was limited; as for one administration, multiple pituitaries of slaughtered bovines were required [1]. This changed in the 1980s when biotechnology offered the opportunity to produce recombinant bovine somatotropin (rbST) [2], enabling unlimited production of the growth hormone and therefore its use on a large scale. Currently, its use for milk production enhancement in dairy cattle is licensed in the USA and other countries, with no restriction to the residue levels. In Europe, there is concern about the effects of rbST on the well-being of the animals and consumers concern related to elevated IGF-1 hormone levels in milk. Because of this, rbST use is banned in Europe [3]. The ban requires methods to detect rbST abuse, and several analytical strategies have been reported [4]. Development of methods to detect rbST abuse is a major challenge due to: (i) the low concentrations in blood, (ii) its sequence similarity with the endogenous hormone bST (only differing in the N-terminal amino acid) and (iii) the strong fluctuations of levels of bST in serum [5, 6]. For these reasons, adequate screening methods aiming at rbST itself, at relevant levels, have failed to be developed so far. Recently, a polyclonal antibody highly selective for rbST was produced and used in an ELISA format [7]. This assay, however, still lacks the required sensitivity to detect rbST in serum at relevant levels by factors of 10 to 100. To overcome the problems in detecting rbST directly, indirect screening methods focussed on detection of rbST-related biomarkers instead of rbST itself [8, 9]. A multiplex screening approach developed by Ludwig et al. [8] provided a serum biomarker profile which correctly predicted rbST use in 95 % of treated dairy cows. The number of true positives met the detection capability (CCβ) requirements of Commission Decision 2002/657 [8, 10]. According to this decision, for positive findings (i.e. suspect samples) of a forbidden substance in a screening assay, additional instrumental analysis is needed to confirm its identity. Subsequent confirmation of forbidden substances, like rbST, can be accomplished by targeted LC-MS/MS analysis [11]. So far, only one confirmatory method for rbST in serum of dairy cows has been described in literature [5]. This method was able to detect rbST in cow serum at concentrations from 4 to 10 ng mL^−1^ during a detection window from 4.5 h to 4 days after treatment [5]. However, the method requires a large serum volume of 4 mL and an extensive sample preparation for cleanup [5]. Furthermore, detection of rbST until 4 days after administration is not sufficient to be able to detect the use of slow-releasing rbST formulas applied only biweekly, according to treatment schedules [12]. Several studies showed that overall bST serum levels (i.e. bST and rbST combined) after rbST treatment in dairy cows range from 1.5 to 45 ng mL^−1^ [6, 13–15]. Consequently, to enable detection of rbST during both the (biweekly) treatment period and the period after treatment, detection methods need sensitivity at/or below 1.5 ng mL^−1^. To achieve this sensitivity, selective purification by use of antibodies against rbST is a promising approach. The use of immuno-affinity purification methods have been shown to be very effective for the detection of clinical important proteins in serum [16]. Therefore, it is expected that application of this approach to rbST will offer the sensitivity needed. In general, anti-rbST antibodies need to be coupled to a carrier, after which enrichment of the low abundant rbST from the complex, protein-rich, serum background can be accomplished. Monolith micro-columns are a promising carrier for the antibody because of their low nonspecific binding, in comparison with beads and the intensive contact between the analyte and the antibodies, as shown in literature for IGF-1 enrichment [17]. For rbST in serum samples, selective and sensitive enrichment with monolith micro-columns may be promising as well. After enrichment of rbST from the serum samples, tryptic digestion was applied. The main advantage of tryptic digestion is the improved sensitivity of the peptides in comparison with detection of the whole protein. In this study, we present the development of a sensitive confirmatory method for rbST in serum of dairy cattle (Fig. 1). Performance characteristics were determined in a preliminary in house validation. Applicability is demonstrated by confirmation of rbST at relevant concentrations in serum samples from rbST treated cows.

## Experimental

### Materials

Monsanto rbST standard was obtained from the National Hormone & Peptide Program (NHPP) of Dr. Parlow (Torrance, CA). Elanco rbST was obtained from Elanco (Indianapolis, IN, USA). Lactotropin 500 mg single-dose syringes were purchased from Centro de Tecnologia (Rio de Janeiro, Brazil).

Pierce BCA protein assay, the Finnpipette™ Novus i Multichannel Electronic and monolithic micro-columns (MSIA disposable automation research tips (D.A.R.T.), containing approximately 10 mg packed bed Protein A or Protein A/G) were all purchased from Thermo Fisher Scientific (Rockford, Illinois). Ammonium sulphate, hydrochloric acid, potassium phosphate, sodium hydroxide, sodium phosphate and the ultrasonic cleaner were purchased from VWR International (Amsterdam, The Netherlands). Trypsin, tris(hydroxymethyl)aminomethane, iodoacetamide (IAA), dimethyl sulfoxide (DMSO) and dl-dithiothreitol were purchased from Sigma-Aldrich Chemie (Zwijndrecht, The Netherlands). Methanol and acetonitrile were purchased from Biosolve (Valkenswaard, The Netherlands). Formic acid was purchased from Actu-All chemicals (Oss, The Netherlands). Protein Lobind Tubes (1.5 mL, 2.0 mL) and a table centrifuge model 5810R were obtained from Eppendorf (Hamburg, Germany). The Jouan GR 20-22 ultracentrifuge was obtained from Jouan (Saint-Herblain, France). The Snijder test tube rotator was purchased from Omnilabo International (Breda, The Netherlands). An isotopic-labelled bST peptide AFPAMSLSGLFANAVLR and a synthetic analogue of the rbST peptide MFPAMSLSGLFANAVLR were obtained from Bachem (Bubendorf, Switserland). The LC-column: Kinetex 50 × 2.10 mm I.D. 1.3 μm C_18_ (100 Å) was purchased from Phenomenex (Utrecht, the Netherlands). Bond Elut Plexa 30 mg solid-phase extraction columns were purchased from Agilent Technologies (Amstelveen, The Netherlands). A Zymark TurboVap was purchased from Biotage (Upsala, Sweden).

### Serum samples

Serum samples from two controlled animal treatment studies were used. In the first animal treatment study, serum samples were obtained from one 3-year-old dairy cow (a) treated twice with subcutaneous injections of 500 mg Lactotropin. This treatment was part of a sequential Lactotropin-steroid treatment schedule existing of three compounds in total. Of each compound, two subcutaneous injections were administered with 1 week interval. After each treatment, an adaptation period of 2 weeks was taken into account. Blood samples were collected daily during the week after each treatment. The second animal treatment study was according to commonly used rbST treatment conditions as recommended by the manufacturer: An adaptation period of 2 weeks was taken into account, and then the cow was treated every second week with 500 mg rbST, according to manufacturers’ guidelines. Serum samples were obtained from one 3-year-old dairy cow (b).

After blood collection, the blood sample was placed at room temperature for 4 h to coagulate. After coagulation, the samples were centrifuged for 10 min at 3000×*g*, and serum was collected and stored at −20 °C until further use. The experimental procedure was authorized by the ethical committee of ID-DLO in Lelystad, the Netherlands.

### Preparation of polyclonal antiserum

The preparation of polyclonal antiserum against Elanco rbST was described before by Heutmekers et al. [18]. Briefly, a New Zealand White rabbit (no. 58) was immunized with Elanco rbST at the Centre for Small Laboratory Animals in Wageningen, the Netherlands. Blood was obtained at various moments during the entire treatment period and serum was collected. The sera collected over the total treatment were pooled and stored at −80 °C for further use.

### Ammonium–sulphate purification of polyclonal antiserum

To concentrate the antibodies from the antiserum and to remove abundant proteins, first, the combined rabbit antiserum no. 58 was diluted three times with PBS (154 mM NaCl, 5.39 mM Na_2_HPO_4_, 1.29 mM KH_2_PO_4_, pH 7.4). Then, slowly, under constant stirring, an equal amount of saturated ammonium sulphate was added. Next, this solution was left at room temperature for 30 min without stirring. Subsequently, the solution was ultra-centrifuged for 10 min at 10,000×*g*, the supernatant was discarded and the pellet was re-suspended in PBS to restore the starting serum volume. Finally, the re-suspended pellet was dialyzed against PBS for 24 h. The protein concentration was 6.6 mg mL^−1^ determined by the BCA protein assay according to the manufacturer’s protocol.

### Immobilization of purified antibodies on protein A and protein A/G monolithic micro-columns followed by rbST enrichment

Polyclonal anti-rbST ammonium–sulphate purified antibodies were immobilized to monolithic micro-columns loaded with, respectively, protein A or protein A/G by affinity binding. The pipet tips containing the protein A and protein A/G monolithic micro-columns were placed on the Finnpipette® Novus i multichannel, which is an automated device having a repetitive cycling function. First, the monolithic micro-columns were washed ten times with 150 μL 20 mM phosphate buffer pH 7. Adherent solution was removed from the column by air pressure. Next, 75 μL 0.1 mg mL^−1^ of the ammonium–sulphate-precipitated polyclonal anti-rbST antibodies, resuspended in 20 mM phosphate buffer pH 7, was transferred over the column 1000 times. After the last cycle, adherent solution was removed from the column by air pressure. To remove unbound antibodies, the monolithic micro-columns were washed twice by 10 cycles of 150 μL 20 mM phosphate buffer pH 7. It took 50 min to obtain these freshly prepared monolith micro-columns immobilized with ammonium–sulphate-precipitated polyclonal anti-rbST antibodies, which were directly used for rbST enrichment.

For immuno-enrichment, 1 mL serum sample or spiked serum was diluted with 1 mL 20 mM phosphate buffer pH 7. The sample was transferred 2000 times (300 μL per cycle) through the anti-rbST-immobilized monolithic micro-column. The adherent sample was removed from the column by air pressure. To remove the remaining unbound sample, the monolithic micro-columns were washed ten times with 150-μL portions of 20-mM phosphate buffer. The captured rbST was then eluted from the monolith micro-column by 50 μL 200 mM NaOH (20 μL per cycle, 150 times). The eluate was collected, and 50 μL 50 mM Tris pH 7.9 was added before further use (final pH > 10). Eluates were stored at −20 °C until tryptic digestion. The enrichment procedure took 4.5 h.

### Digestion and cleanup of the immuno-affinity-purified extract

For digestion of the proteins with trypsin, the pH of the obtained solution was adjusted to 8–8.5 with 1 M HCl. After addition of 5 μL 45 mM dl-dithiothreitol (DTT), to reduce sulphur bridges, the solution was mixed and incubated for 30 min at 37 °C. The solution was cooled down to room temperature, and 5 μL 0.1 M iodoacetamide was added for methylation of the cysteine residues. The solution was mixed on a vortex and incubated for 30 min at room temperature in the dark. For protein digestion, 5 μg trypsin in 1 mM HCl (pH < 3) was added, followed by 20 μL acetonitrile, mixed by vortex and incubated 1 h at 37 °C. Digestion was stopped by the addition of 0.9 mL 5 % formic acid. Then, an internal standard solution containing isotope-labelled bST peptide was added. This peptide is a replicate of the 17 amino acids of the N-terminal end of endogenous bST (AFPAMSLSGLFANAVLR) and only differs in the N-terminal amino acid from the rbST incorporation of alanine–^13^C_6_^15^N_4_, resulting in a total mass increase of 10 Da. The digest was concentrated on an Agilent Bond Elut Plexa SPE column (60 mg): After conditioning the column with 1 mL methanol and 1 mL 5 % formic acid in water, the sample was applied onto the column. Then, the column was washed with 1 mL 10 % acetonitrile and the sample was eluted with 0.5 mL water/acetonitrile/formic acid (25:70:5, *v/v/v*). The eluate was collected in 50 μL DMSO and evaporated to approximately 60 μL on a TurboVap at 55 °C under 10 psi N_2_. Please note that the exact volume is not critical due to the use of an internal standard. After cooling to room temperature, 25 μL 5 % formic acid was added. The sample was mixed and transferred to an LC injection vial. The digestion and concentration procedure took in total 3 h.

### LC-MS/MS analysis

Analysis was performed using an I-Class UPLC system connected to a Xevo TQS mass spectrometer Waters (Manchester, UK). Thirty microliters from the final extract was analysed by UHPLC-MS/MS in multiple reaction monitoring (MRM) mode. The chromatographic separation was performed on a Kinetex 50 × 2.10 mm I.D. 1.3 μm C_18_ (100 Å) column. The flow rate was set at 0.5 mL min^−1^. A gradient was used starting with 75:25 (*v*/*v*) water/acetonitrile for the first 30 s, increasing to 70:30 (*v/v*) water/acetonitrile in the next 3 min. Then, the column was washed for half a minute with 100 % acetonitrile. Total run time was 6 min 30 s. The mass spectrometer was operated in the positive ion ESI-MS/MS mode. Ion transitions *m/z* 913.1 > 774.1 and *m/z* 913.1 > 1047.6 were measured to detect the rbST specific N-terminal peptide with amino acid sequence, MFPAMSLSGLFANAVLR, after tryptic digestion [19]. To check the retention time of this N-terminal rbST peptide of interest, a synthetic analogue of the rbST peptide was injected at the beginning and the end of each series. For the bST internal standard, the transition *m/z* 888.1 > 779.13 was followed.

### In-house method validation

The decision limit CCα and the detection capability CCβ were determined according to the calibration procedure conform Commission Decision 2002/657/EC. Calculation of the concentration was performed by constructing a linear calibration curve of the response factor (peak area ratio of rbST fragment and internal standard) vs the concentration (expressed as absolute amount rbST protein). For intra-assay variation, four identical rbST spiked serum samples of respectively 2 and 10 ng mL^−1^ rbST in serum were analysed in parallel. Variation was determined and expressed as the percentage of the average. For determination of inter-assay variation, the spiked serum samples of 2 and 10 ng mL^−1^ were prepared, enriched with monolith micro-columns and measured on three different days. Variation was determined and expressed as the percentage of the average. For recovery of the immuno-affinity isolation, rbST-spiked serum samples were analysed and compared to rbST calibration curve in sodium hydroxide, as the latter is compatible with the elution conditions after immune-affinity rbST enrichment.

## Results and discussion

### Optimization rbST immuno-affinity enrichment

For the enrichment of the low abundant rbST from serum of dairy cattle (Fig. 1), two monolith micro-columns were compared: a monolith micro-column prepared with protein A and a monolith micro-column prepared with protein A/G. Both protein A and protein A/G have a high affinity for polyclonal rabbit antibodies [17] and are expected to strongly interact with the rabbit anti-rbST used in this study. After rabbit anti-rbST immobilization on both monolith micro-columns, the protein A monolith micro-column was able to capture 10–20 % more rbST from spiked serum samples compared with the protein A/G monolith micro-column. Therefore, the protein A monolith micro-column was used for further optimization steps.

To obtain the highest recovery of rbST, the number of pipetting cycles, pipetting speed, antibody immobilization concentration and elution conditions were investigated with spiked serum samples. The number of pipetting cycles was determined to be 2000, taking both the efficiency of enrichment and time into consideration. The pipetting speed was found to be of great importance for both immobilization of the antibody on the monolith micro-column and for rbST capture from serum samples. To effectively immobilize the polyclonal anti-rbST antibody onto the monolith micro-column, the solution had to be transferred through the column with slowest pipetting rate as practically possible (approximately 63 μL s^−1^). Otherwise, immobilization of the antibody was not sufficient and no rbST was captured. The same result was obtained for the transfer of serum samples over the monolith micro-column to enable rbST capture: rbST could only be detected when the serum was transferred slowly through the micro-column (approximately 77 μL s^−1^).

**Fig. 2:**
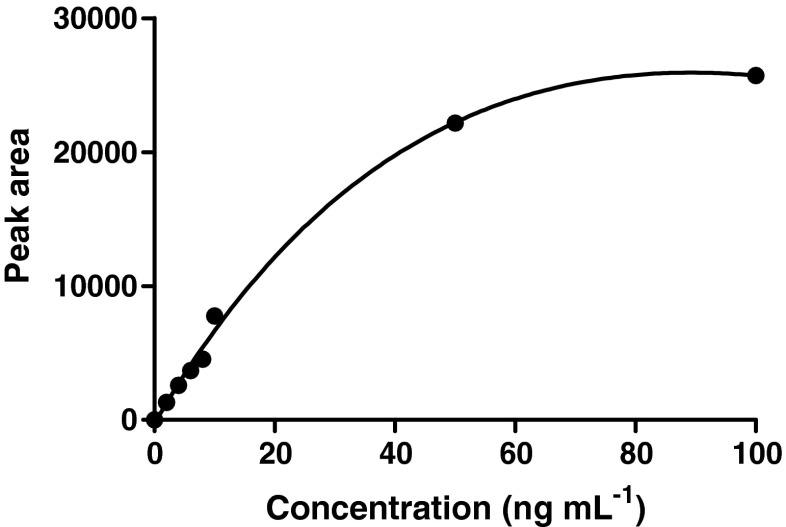
Peak areas obtained with LC-MS/MS transition *m/z* 913.1 > *m/z* 774.13 after enrichment of rbST in spiked serum samples at: 0, 2, 4, 6, 8, 10, 50 and 100 ng mL^−1^. The observation of a plateau beyond 50 ng mL^−1^ indicates saturation of the binding sites of the micro-columns

Next, the concentration of anti-rbST antibody used for immobilization to the monolith micro-columns was optimized, aiming for the highest yield of rbST after enrichment. For this, anti-rbST was applied to the column with concentrations of 0.1, 0.07, 0.04 and 0.01 mg mL^−1^ in 20 mM phosphate buffer pH 7. The immobilization concentration of 0.1 mg mL^−1^ showed best rbST capture capacity respectively 2, 3 and 7 times more rbST was captured using 0.1 mg mL^−1^ compared with the other concentrations. Higher concentrations of the antibody were not tested as immobilization with 0.1 mg mL^−1^ antibody was capable to enrich rbST in serum at concentrations in the low nanogram per milliliter range, sensitive enough for incurred serum samples. The binding sites of the micro-columns prepared under these conditions were found to be saturated at a serum rbST concentration of >50 ng mL^−1^, which is at least 50 times higher than the expected levels of rbST in treated cows (Fig. 2).

For the elution of captured rbST, conditions compatible with the subsequent trypsin digestion are preferred to simplify the workflow. Trypsin digestion compatible buffers are for instance tris and ammonium bicarbonate. These buffers were tested in different molarities for their capacity to elute rbST from the monolith micro-columns. Unfortunately, these conditions are too mild, only eluting 10 % or less rbST from the monolith micro-columns in comparison with harsher elution conditions. A solution of 200 mM NaOH was found to be most effective for elution of rbST from the micro-columns. After elution, a tris solution was added to obtain a trypsin compatible solution. In Fig. 1, an overview of the entire workflow developed is given. In short, after enrichment of rbST with monolith micro-columns from serum supernatant, tryptic digestion yields 20 different peptides. The endogenous and recombinant protein differs by one amino acid located at the N-terminal side of the protein. In case of rbST, an alanine is replaced by a methionine. To discriminate between these two forms, the N-terminal peptide is analysed. To be sure that the detected N-terminal peptide is specific, a blast computation was performed at the SIB using the BLAST network service [20]. There were no other peptides found containing the same amino acid sequence as the detected peptide. The addition of the isotope-labelled N-terminal peptide of bST as an internal standard allows to correct for sample cleanup losses after tryptic digestion.

### Method performance characterization

To characterize the method performance, the developed method was partly validated in-house as a quantitative confirmatory method according to Commission Decision 2002/657/EC [10]. The parameters considered were the decision limit (CCα), the detection capability (CCβ), the intra-assay variation, the inter-assay variation and the recovery. Ion ratio of the measured transition determined for samples from the matrix matched sample (MMS) series and samples from rbST-treated animals were all within the ion ratio limits, as described in the Commission Decision 2002/657/EC [10].

Important parameters for performance characterization are (i) the decision limit (CCα), the limit at and above which it can be concluded, with an error probability of α, that a sample is non-compliant; (ii) the detection capability (CCβ), the smallest content of the substance that may be detected, identified and quantified in a sample with an error probability of β; (iii) the intra-assay variation; (iv) the inter-assay variation; and (v) the recovery. In the case of substances for which no permitted limit has been established, the detection capability is the lowest concentration at which a method is able to truly detect contaminated samples with a statistical certainty of 1 − β.

**Fig. 3:**
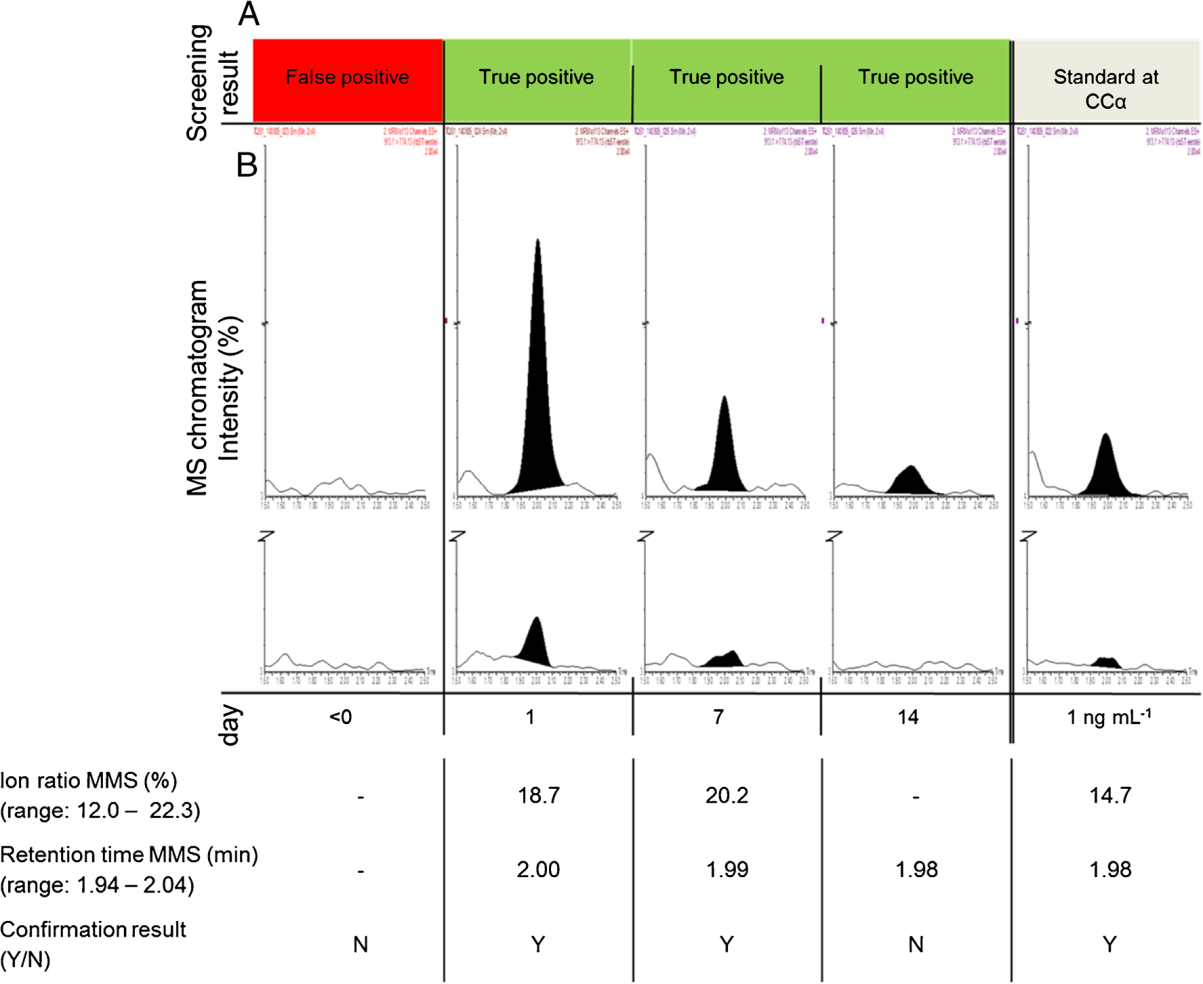
*A Top bar* shows the results of the screenings analysis, *B* LC-MS/MS confirmation of biomarker screening results. Chromatograms of transition *m/z* 913.1 > *m/z* 774.13 and *m/z* 913.1 > *m/z* 1047.6 after enrichment of rbST in serum in (*from left to right*): a serum sample prior to treatment (day 0), a serum sample taken the first day of treatment (determined to contain 9.0 ng mL^−1^ rbST), a serum sample taken 7 days after treatment (determined to contain 1.9 ng mL^−1^ rbST), a serum sample taken 14 days after treatment (determined to contain < 1 ng mL^−1^) and a serum sample spiked with 1 ng mL^−1^ rbST. All *y*-*axes* are scaled to the serum sample of the first day of treatment. Below the chromatogram, the compliance with the ion ratio and retention time criteria is indicated (the ion ratio and retention time interval as determined according to the 2002/657/EC are given in *brackets*)

Data obtained from the validation study are presented in Table 1. The CCα and CCβ were determined to be 0.8 and 1.6 ng mL^−1^, respectively. From additional samples spiked with rbST in concentrations of 0.25, 0.5 and 1 ng mL^−1^, it was concluded that the obtained CCα and CCβ are realistic, as the 0.5 and 1 ng mL^−1^ samples still meet the ion ratio criteria (see also the reconstructed ion chromatogram of the rbST-spiked serum sample at 1 ng mL^−1^ in Fig. 3). Moreover, in 75 % of blank serum samples spiked at CCα (0.8 ng mL^−1^), rbST presence was confirmed, which is actually better than the >50 % at CCα level as required by 2002/657/EC.

**Table 1:** In-house validation study to characterize the method performance using monolith micro-columns loaded with protein A, tryptic digestion and LC-MS/MS analyses

Validation results		
CCα		0.8 ng mL^−1^
CCβ		1.6 ng mL^−1^
Intra-assay variation	2 ng mL^−1^	12 % (*n* = 4)
	10 ng mL^−1^	9 % (*n* = 3)
Inter-assay variation	2 ng mL^−1^	15 % (*n* = 3)
	10 ng mL^−1^	10 % (*n* = 3)

Intra- and inter-assay variations were determined for rbST in serum at a concentration of 2 and 10 ng mL^−1^, in accordance with expected serum concentrations. Variation was expected to be higher at 2 ng mL^−1^ compared with 10 ng mL^−1^. Although this difference was indeed observed in both intra- and inter-assay variation, intra-assay variation was found to be only 12 % at 2 ng mL^−1^ and 9 % at 10 ng mL^−1^ rbST in serum (Table 1). It should be noted that for 10 ng mL^−1^, one of the four data points was removed as outlier due to the fact that the internal standard was not added correctly.

**Fig. 4:**
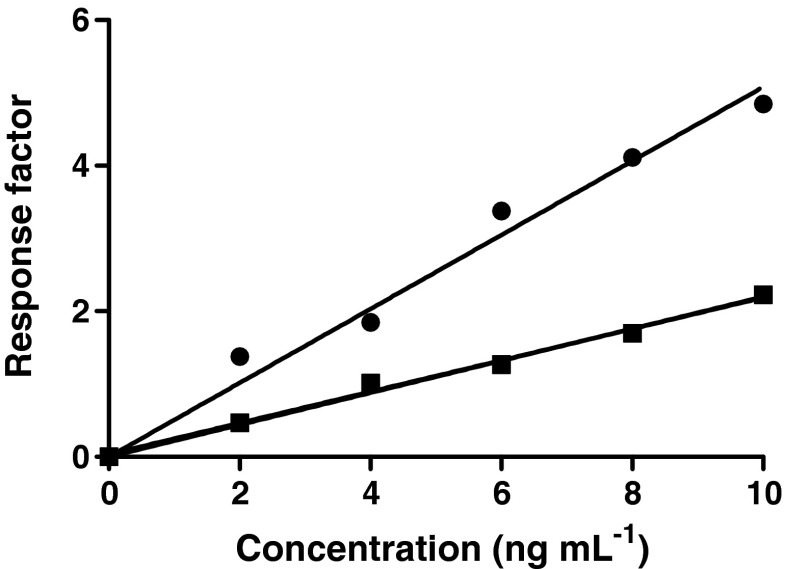
Calibration curves with respectively 0, 2, 4, 6, 8 and 10 ng mL^−1^ of rbST spiked in NaOH (*black circle*) to simulate enrichment elution conditions and after immuno-affinity enrichment of fortified serum samples (*black square*)

Recovery was determined by comparison of calibration curves prepared in either elution solvent or by fortification of serum. The graphs in Fig. 4 shows that not all rbST from the fortified serum samples will be captured and eluted using the monolith micro-columns. Comparison of the response factors of the two calibration curves suggests a recovery during immuno-enrichment of approximately 50 % for concentrations up to 10 ng mL^−1^. For the two highest concentrations, 50 and 100 ng mL^−1^, the recovery of rbST dropped to 25 and 17 %, respectively, due to saturation of the binding sites of the monolith micro-column (Fig. 2). Even though not all rbST is recovered, the repeatability and reproducibility data in Table 1 are fit for purpose and the sensitivity required for effective control is reached.

To test the stability of the trypsin-digested serum, samples were stored for 2 weeks at 4–8 °C. Comparison of the samples before and after storage showed a decrease in peak area of approximately 50 % for all samples (results not shown). The decrease in intensity of the measured N-terminal peptide can be explained by its instability and tendency to adsorb to glassware [21]. Although 50 % is a significant loss at 4–8 °C, it did not obstruct measurement and quantification of rbST in the extracts. It is therefore advised to store digested samples and matrix-matched calibrants at 4–8 °C as short as possible prior to analysis.

Detection of rbST in bovine serum has already been presented before by Le Breton et al. [5]. In that work, the lowest presented rbST concentration in bovine serum was 3 ng mL^−1^ (data acquired on only one transition). To reach that level, an extensive sample preparation of multiple precipitation steps and overnight digestion was needed. In contrast, the method presented in this study shows high sensitivity (CCα of 0.8 ng mL^−1^) and the confidence of data acquisition of two ion transitions. Note that using a single-ion transition, rbST in bovine serum with a concentration of 0.25 ng mL^−1^ could even be detected. Moreover, sample preparation is less extensive, less laborious and semi-automated, and tryptic digestion required only 1 h.

### rbST analysis in serum of rbST-treated cows

To investigate applicability of the developed method to real-life samples, serum samples from two different animal experiments were analysed. As a proof of principle, from the first animal experiment, 17 serum samples of one treated cow were analysed: two serum samples prior to treatment (*t* = <0 days), one serum sample for each day after the first and second treatment (*t* = 1–14 days) and one sample 3 weeks after the second treatment (*t* = 29 days). This allowed exploring the detectability of rbST during and after the treatment period. Obtained rbST concentrations, corrected for incomplete recovery, are given in Table 2 and show that rbST can be detected from the first day after the first treatment until 7 days after the second treatment. Only in one serum sample, taken on day 2, no rbST seemed to be present. It is not clear why no rbST was detected in the sample that day. The highest concentration of rbST (21.4 ng mL^−1^) was detected on the first day after the first treatment. On the first day after the second treatment, an increase in rbST was observed as well, although less apparent. In general, a daily variation in rbST concentration was observed and rbST concentrations decreased only slowly. This suggests the ability of the slow-releasing formulae to release rbST slowly to the blood circulation, with a peak on the first day(s), and maintain a minimum level. Twenty-one days after the last treatment, no rbST could be detected anymore, which is in good agreement with the need of a two-weekly treatment, as advised by the manufacturer [12]. This treatment schedule was applied in the second animal study. Analysis of samples from this study gives insight in the ability of the method to detect rbST use under realistic treatment conditions. Four serum samples were analysed: one serum sample prior to treatment (*t* = <0 days) and serum samples taken after 1 day (*t* = 1 day), 1 week (*t* = 8 days) and 2 weeks (*t* = 14 days) after the third treatment. In addition, three additional blank serum samples from other animals were analysed.

**Table 2:**
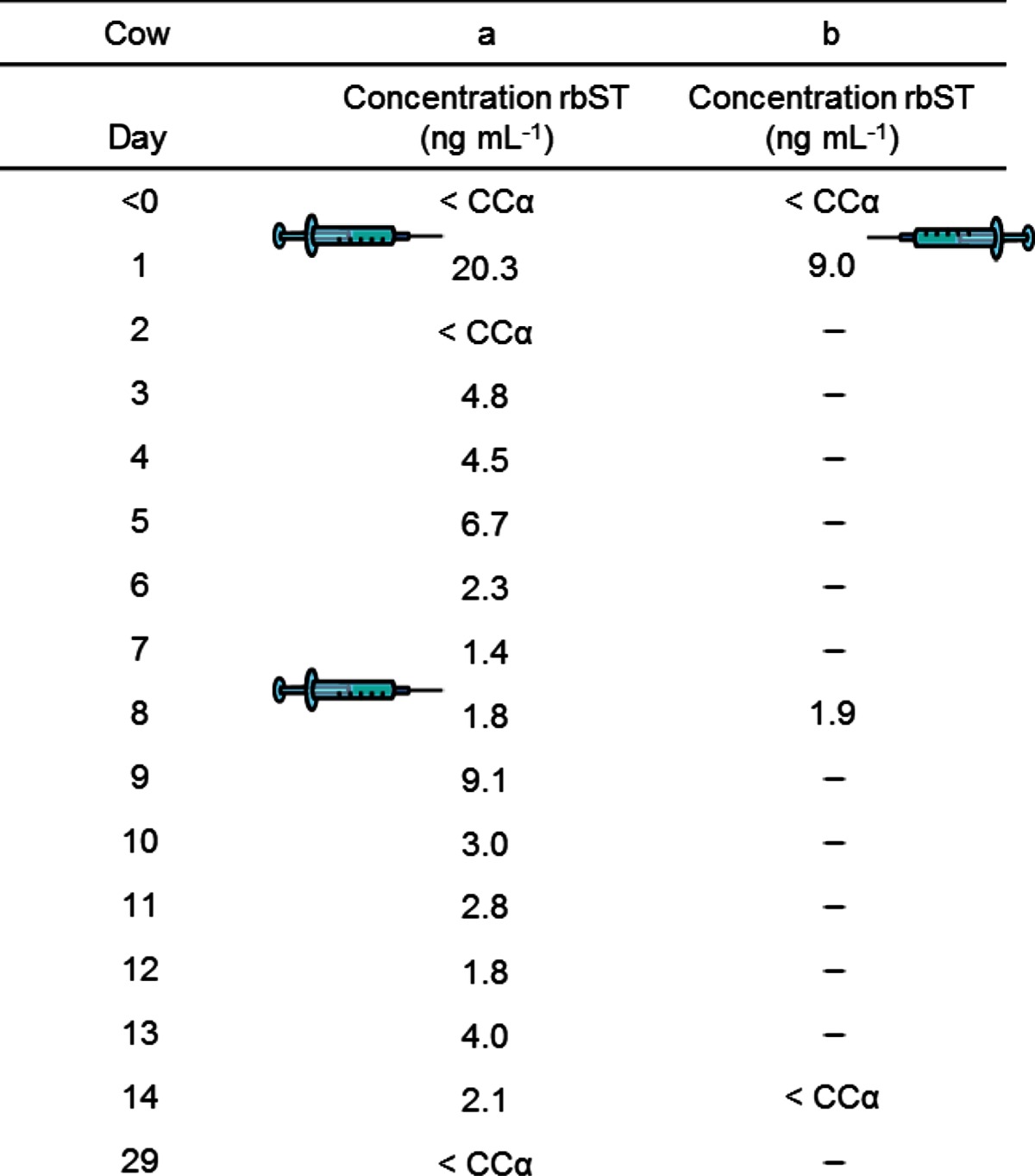
RbST concentrations found in serum samples of two dairy cows, a and b treated with rbST, quantified versus MMS

Syringes (left side for cow a; right side for cow b) show days of treatment with rbST in slow releasing formulae

“–” not determined

No rbST was detected in any of the five blank serum samples, and no interferences are observed that could lead to false-positive findings. This implies that the method is very specific, though analysis of more non-treated animals is needed to statistically prove these findings. From the treated cow (b), the trend of the determined rbST concentrations is similar to the first animal experiment. The highest concentration was found on the first day after administration, and rbST could be detected until 1 week after administration (see Table 2 and Fig. 3). The difference in concentration on the first day after treatment between the two cows from the two animal experiments can be explained by natural variation in response, which should be investigated by analysis of more treated animals. For the serum sample taken 2 weeks after rbST administration, just prior to the following administration, a peak is clearly visible at transition *m/z* 993.1 > *m/z* 774.13 and suggests the presence of rbST (Fig. 3). However, the concentration is lower than the CCα of the method, and for confirmation of rbST according to Commission Decision 2002/657/EC, a concentration larger than CCα and a peak at a second transition are necessary. Further enhancement of the sensitivity may be obtained by increasing the sample volume. It is expected that by this adjustment, rbST presence can not only be detected but also confirmed up to 14 days after rbST administration. The results compare favourably with previous methods [5], in which rbST was only detected until 4 days after treatment.

### Confirmation of positive screening results

Control strategies in food and feed safety often include two steps: First, samples are screened in order to obtain a fast indication of the suspect samples, thereby reducing the number of samples, as samples with a negative result will not be investigated further. The second step in the control strategy is the confirmation of positive samples conform Commission Decision 2002/657 with additional (analytical) methods [10]. This two-step strategy was applied to the four samples from the second animal study. The samples were previously screened by Ludwig et al. [8] by a multiple protein biomarker assay, where four different biomarker proteins were measured simultaneously. The serum samples were selected for analysis with the LC-MS/MS method as they were considered suspicious for rbST in the screening assay (Fig. 3a). The sample taken prior to treatment (*t* = <0 days) was screened suspect, which is obviously unlikely and might serve as a false-positive case. Analysis of the serum sample prior to treatment (*t* = <0 days) with our confirmatory method showed that, indeed, despite the positive screening result, no rbST-specific peptide could be detected and rbST was below our detection limit (Fig. 3b). It can therefore be concluded that the screening result of this sample, taken before treatment, is false positive and underlines the necessity of confirmatory methods. The false-positive screening result was most likely due to the apparent presence of rbST-induced antibodies as was observed in less than 5 % of the untreated cows and is most likely the result of non-specific interactions of other antibodies or proteins in the screening assay [22].

For the other three serum samples, taken during rbST treatment according to the treatment schedule, screening results were found to be true positive. As shown in Fig. 3b by the chromatograms of transition *m/z* 913.1 > *m/z* 774.13 and *m/z* 913.1 > *m/z* 1047.6, samples taken during the rbST treatment all showed rbST presence.

Samples taken after treatment of dairy cows with rbST (*t* = >0 days), which were found positive in the screenings assay, were confirmed for the presence of rbST with the developed confirmatory LC-MS/MS method (Fig. 3). In addition, the need of a reliable confirmatory method for samples found positive during screening was proven by the example of a false-positive screening result that could only be identified by the LC-MS/MS analysis. Please note that no explicit confirmation criteria have been established yet for protein and/or peptide analysis by targeted MS/MS.

## Conclusion

In this study, a novel approach to pinpoint rbST abuse has been developed based on rbST enrichment by immuno-affinity on monolith micro-columns. High sensitivity is reached with a CCα of 0.8 ng mL^−1^. The intra- and inter-assay variations were determined to be <10 % at 10 ng mL^−1^ and <18 % at 2 ng mL^−1^. Applicability of the confirmatory method was demonstrated by analysis of serum samples from a treated animal for which positive screening results were obtained. With the developed approach, we quantify rbST with a detection window of the total treatment period of 14 days and confirm its presence for over 1 week after treatment. It is therefore that, for the first time, to the best of our knowledge, an approach is presented that successfully proofs rbST abuse under commonly used treatment conditions.
